# 4-Bromo­benzoic acid–6-(4-bromo­phen­yl)-3-methyl-1,2,4-triazolo[3,4-*b*][1,3,4]thia­diazole (1/1)

**DOI:** 10.1107/S1600536812012184

**Published:** 2012-03-24

**Authors:** Kamini Kapoor, Vivek K. Gupta, Satya Paul, Seema Sahi, Rajni Kant

**Affiliations:** a X-ray Crystallography Laboratory, Post-Graduate Department of Physics & Electronics, University of Jammu, Jammu Tawi 180 006, India; b Department of Chemistry, University of Jammu, Jammu Tawi 180 006, India

## Abstract

In the title 1:1 co-crystal, C_10_H_7_BrN_4_S·C_7_H_5_BrO_2_, the triazolothia­diazole system is approximately planar [with a maximum deviation of 0.030 (4) Å] and forms a dihedral angle of 8.6 (1)° with the bromo­phenyl ring. In the carb­oxy­lic acid mol­ecule, the carboxyl group is rotated by 6.4 (3)° out of the benzene ring plane. The crystal structure features O—H⋯N and C—H⋯O hydrogen bonds, π–π stacking inter­actions [centroid–centroid distances = 3.713 (2), 3.670 (2) and 3.859 (3) Å] and short S⋯N [2.883 (4) Å] contacts.

## Related literature

For the biological activity of triazole derivatives, thia­diazo­les and triazolothia­diazole compounds, see: Chaturvedi *et al.* (1988 ▶); Holla *et al.* (2003 ▶); Bhat *et al.* (2004 ▶); Bekircan & Bektas (2006 ▶); Shawali & Sayed (2006 ▶); Mathew *et al.* (2007 ▶); Karthikeyan *et al.* (2007 ▶); Zhou *et al.* (2007 ▶). For related structures, see: Dinçer *et al.* (2005 ▶); Arshad *et al.* (2009 ▶); Jia *et al.* (2011 ▶). For bond-length data, see: Allen *et al.* (1987 ▶).
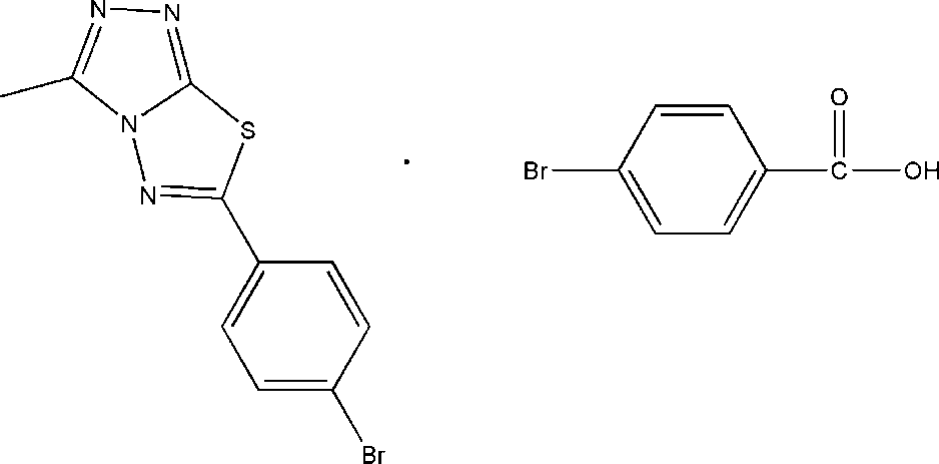


## Experimental

### 

#### Crystal data

C_10_H_7_BrN_4_S·C_7_H_5_BrO_2_
*M* *_r_* = 496.19
Triclinic, 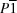

*a* = 7.7592 (3) Å
*b* = 8.0634 (4) Å
*c* = 14.9076 (7) Å
α = 94.090 (4)°
β = 92.961 (3)°
γ = 99.326 (4)°
*V* = 916.13 (7) Å^3^
*Z* = 2
Mo *K*α radiation
μ = 4.56 mm^−1^
*T* = 293 K
0.3 × 0.2 × 0.2 mm

#### Data collection

Oxford Diffraction Xcalibur Sapphire3 diffractometer
Absorption correction: multi-scan (*CrysAlis PRO RED*; Oxford Diffraction, 2010 ▶) *T*_min_ = 0.581, *T*_max_ = 1.000
8264 measured reflections
3594 independent reflections
2254 reflections with *I* > 2σ(*I*)
*R*_int_ = 0.036

#### Refinement

*R*[*F* ^2^ > 2σ(*F* ^2^)] = 0.048
*wR*(*F* ^2^) = 0.116
*S* = 1.01
3594 reflections
236 parameters
H-atom parameters constrained
Δρ_max_ = 0.43 e Å^−3^
Δρ_min_ = −0.51 e Å^−3^

### 

Data collection: *CrysAlis PRO CCD* (Oxford Diffraction, 2010 ▶); cell refinement: *CrysAlis PRO CCD*; data reduction: *CrysAlis PRO RED* (Oxford Diffraction, 2010 ▶); program(s) used to solve structure: *SHELXS97* (Sheldrick, 2008 ▶); program(s) used to refine structure: *SHELXL97* (Sheldrick, 2008 ▶); molecular graphics: *ORTEP-3* (Farrugia, 1997 ▶); software used to prepare material for publication: *PLATON* (Spek, 2009 ▶).

## Supplementary Material

Crystal structure: contains datablock(s) I, global. DOI: 10.1107/S1600536812012184/bh2421sup1.cif

Structure factors: contains datablock(s) I. DOI: 10.1107/S1600536812012184/bh2421Isup2.hkl

Supplementary material file. DOI: 10.1107/S1600536812012184/bh2421Isup3.cml

Additional supplementary materials:  crystallographic information; 3D view; checkCIF report

## Figures and Tables

**Table 1:** Hydrogen-bond geometry (Å, °)

*D*—H⋯*A*	*D*—H	H⋯*A*	*D*⋯*A*	*D*—H⋯*A*
O24—H24⋯N2^i^	0.82	1.87	2.674 (4)	169
C9—H9*A*⋯O23^i^	0.96	2.48	3.393 (6)	159

Symmetry code: (i) 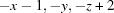.

**Fig. 1.**
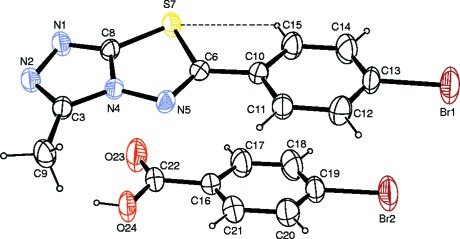
ORTEP view of the asymmetric unit of the title cocrystal, with thermal ellipsoids drawn at the 40% probability level. H atoms are shown as small spheres of arbitrary radii.

**Fig. 2.**
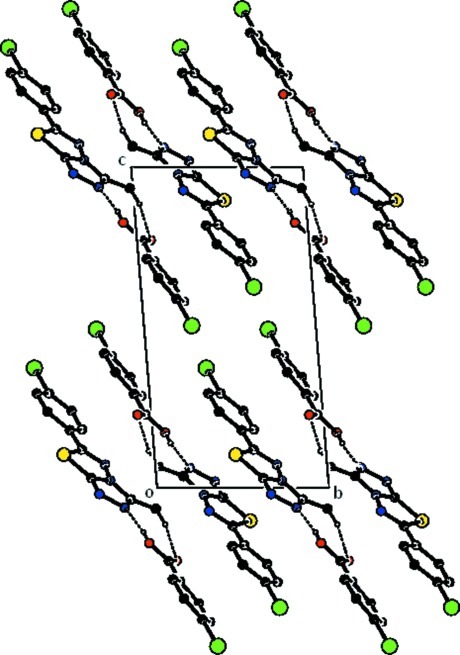
The packing arrangement of molecules viewed down the a axis. The broken lines show the intermolecular O—H···N and C—H···O interactions.

## supplementary crystallographic information

### Comment

Derivatives of 1,2,4-triazole possess a wide spectrum of biological activity, such as anticancer, anticonvulsant, analgesic, antibacterial, anthelmintic, antitubercular and anti-inflammatory activities (Holla *et al.*, 2003; Bekircan & Bektas, 2006; Zhou *et al.*, 2007). Similarly 1,3,4-thiadiazoles were also found to possess antitumor, anti-inflammatory, antibacterial, antifungal, anticonvulsant and antitubercular properties (Bhat *et al.*, 2004; Mathew *et al.*, 2007). Thus triazolothiadiazole systems may be viewed as cyclic analogues of two very important components, which often display diverse pharmacological properties. Triazolothiadiazoles obtained by fusing the 1,2,4-trizole and 1,3,4-thiadiazole rings together have been reported to possess similar biological properties (Chaturvedi *et al.*, 1988; Shawali & Sayed, 2006; Karthikeyan *et al.*, 2007). Here we report the crystal structure of the 1:1 cocrystal of a triazolothiadiazole derivative and 4-bromobenzoic acid.

Bond lengths (Allen *et al.*, 1987) and angles in the title compound (Fig. 1) have normal values and also correspond to those observed in related structures (Dinçer *et al.*, 2005; Arshad *et al.*, 2009; Jia *et al.*, 2011). The triazolothiadiazole ring is planar with a maximum deviation of 0.030 (4) Å for atom C6. The plane through the benzene ring forms dihedral angle of 8.6 (1)° with the triazolothiadiazole unit. In the molecular structure, an intramolecular C15—H15···S7 contact leads to the formation of a five-membered ring which is fused with the phenyl ring (Fig. 1).

In the crystal structure of the title compound, intermolecular O—H···N and C—H···O hydrogen bonds (Table 2) link the triazolothiadiazole molecule with 4-bromobenzoic acid (Fig. 2). In addition to these interactions, the crystal structure contains three π–π stacking interactions. The first of these is between the thiadiazole ring and its symmetry-related partner at (-*x*, 1-*y*, -*z*), with a distance of 3.713 (2) Å between the ring centroids, and a perpendicular distance between the rings of 3.468 Å. The second is between the triazole ring and the benzene ring at (-*x*, 1-*y*, -*z*), with a distance of 3.670 (2) Å between the ring centroids and a perpendicular distance between the rings of 3.427 Å. The third is between the benzene rings (C10···C15) and (C16···C21) in the asymmetric unit, with a distance of 3.859 (3) Å between the ring centroids and a perpendicular distance between the rings of 3.599 Å. A short contact distance not listed in tables, yet noteworthy, is S7···N1 with N1 at position (-*x*-1, -*y*+1, -*z*+2), the S···N separation being 2.883 (4) Å, which may cause steric hindrance.

### Experimental

4-Amino-5-mercapto-3-methyl-1,2,4-triazole (0.130 g, 1 mmol) and 4-bromo-β-chlorocinnamic acid (0.261 g, 1 mmol) were stirred in POCl_3_ (3 ml) at 80 °C for 30 min. 6-(4-Bromophenyl)-3-methyl-1,2,4-triazolo[3,4-*b*][1,3,4]thiadiazole was obtained along with 4-bromobenzoic acid after pouring the reaction mixture in crushed ice followed by washing with dilute NaOH. Finally, it was crystallized from methanol, affording white crystals.

### Refinement

All H atoms were positioned geometrically and were treated as riding on their parent atoms, with O—H = 0.82 Å for OH, C—H = 0.93 Å for aromatic H, C—H = 0.96 Å for methyl H, and with *U*_iso_(H_aryl_) = 1.2*U*_eq_(C_aryl_), *U*_iso_(H_methyl_) = 1.5*U*_eq_(methyl C), and *U*_iso_(H24) = 1.5*U*_eq_(O24).

### Crystal data

**Table:**

C_10_H_7_BrN_4_S·C_7_H_5_BrO_2_	*Z* = 2
*M**_r_* = 496.19	*F*(000) = 488
Triclinic, *P*1	*D*_x_ = 1.799 Mg m^−^^3^
Hall symbol: -P 1	Mo *K*α radiation, λ = 0.71073 Å
*a* = 7.7592 (3) Å	Cell parameters from 2731 reflections
*b* = 8.0634 (4) Å	θ = 3.4–28.9°
*c* = 14.9076 (7) Å	µ = 4.56 mm^−^^1^
α = 94.090 (4)°	*T* = 293 K
β = 92.961 (3)°	Block, white
γ = 99.326 (4)°	0.3 × 0.2 × 0.2 mm
*V* = 916.13 (7) Å^3^	

### Data collection

**Table:**

Oxford Diffraction Xcalibur Sapphire3 diffractometer	3594 independent reflections
Radiation source: fine-focus sealed tube	2254 reflections with *I* > 2σ(*I*)
Graphite monochromator	*R*_int_ = 0.036
Detector resolution: 16.1049 pixels mm^-1^	θ_max_ = 26.0°, θ_min_ = 3.4°
ω scans	*h* = −9→9
Absorption correction: multi-scan (*CrysAlis PRO* RED; Oxford Diffraction, 2010)	*k* = −9→9
*T*_min_ = 0.581, *T*_max_ = 1.000	*l* = −18→18
8264 measured reflections	

### Refinement

**Table:**

Refinement on *F*^2^	Primary atom site location: structure-invariant direct methods
Least-squares matrix: full	Secondary atom site location: difference Fourier map
*R*[*F*^2^ > 2σ(*F*^2^)] = 0.048	Hydrogen site location: inferred from neighbouring sites
*wR*(*F*^2^) = 0.116	H-atom parameters constrained
*S* = 1.01	*w* = 1/[σ^2^(*F*_o_^2^) + (0.0424*P*)^2^ + 0.2229*P*] where *P* = (*F*_o_^2^ + 2*F*_c_^2^)/3
3594 reflections	(Δ/σ)_max_ = 0.001
236 parameters	Δρ_max_ = 0.43 e Å^−^^3^
0 restraints	Δρ_min_ = −0.51 e Å^−^^3^
0 constraints	

### Fractional atomic coordinates and isotropic or equivalent isotropic displacement parameters (Å^2^)

**Table:**

	*x*	*y*	*z*	*U*_iso_*/*U*_eq_	
Br1	0.48693 (7)	0.65776 (8)	0.62657 (4)	0.0850 (3)	
Br2	0.15874 (8)	0.27859 (8)	0.50571 (4)	0.0967 (3)	
N1	−0.4623 (4)	0.3387 (4)	1.0195 (2)	0.0462 (9)	
N2	−0.4261 (4)	0.1982 (4)	1.0619 (2)	0.0445 (9)	
C3	−0.2777 (5)	0.1581 (5)	1.0362 (3)	0.0397 (10)	
N4	−0.2150 (4)	0.2704 (4)	0.9772 (2)	0.0352 (8)	
N5	−0.0694 (4)	0.2926 (4)	0.9277 (2)	0.0375 (8)	
C6	−0.0778 (5)	0.4209 (5)	0.8811 (3)	0.0340 (9)	
S7	−0.25764 (12)	0.52658 (14)	0.89676 (7)	0.0435 (3)	
C8	−0.3311 (5)	0.3782 (5)	0.9699 (3)	0.0360 (9)	
C9	−0.1926 (5)	0.0180 (5)	1.0658 (3)	0.0554 (13)	
H9A	−0.2529	−0.0311	1.1146	0.083*	
H9B	−0.0728	0.0603	1.0855	0.083*	
H9C	−0.1970	−0.0661	1.0164	0.083*	
C10	0.0567 (5)	0.4805 (5)	0.8205 (3)	0.0361 (9)	
C11	0.2088 (5)	0.4096 (5)	0.8184 (3)	0.0440 (10)	
H11	0.2251	0.3262	0.8565	0.053*	
C12	0.3355 (5)	0.4618 (6)	0.7602 (3)	0.0527 (12)	
H12	0.4359	0.4128	0.7583	0.063*	
C13	0.3124 (5)	0.5858 (6)	0.7056 (3)	0.0480 (11)	
C14	0.1622 (6)	0.6563 (6)	0.7054 (3)	0.0657 (14)	
H14	0.1465	0.7390	0.6667	0.079*	
C15	0.0365 (6)	0.6033 (6)	0.7628 (3)	0.0547 (12)	
H15	−0.0648	0.6511	0.7629	0.066*	
C16	−0.2498 (6)	0.0900 (5)	0.7076 (3)	0.0474 (11)	
C17	−0.2908 (7)	0.1880 (7)	0.6397 (3)	0.0694 (15)	
H17	−0.4023	0.2159	0.6342	0.083*	
C18	−0.1704 (8)	0.2442 (7)	0.5808 (4)	0.0782 (16)	
H18	−0.1989	0.3108	0.5357	0.094*	
C19	−0.0075 (7)	0.2015 (6)	0.5886 (3)	0.0596 (13)	
C20	0.0382 (6)	0.1051 (6)	0.6543 (3)	0.0614 (13)	
H20	0.1500	0.0776	0.6590	0.074*	
C21	−0.0852 (6)	0.0486 (6)	0.7140 (3)	0.0557 (12)	
H21	−0.0560	−0.0181	0.7590	0.067*	
C22	−0.3841 (6)	0.0335 (6)	0.7719 (3)	0.0541 (12)	
O23	−0.5243 (5)	0.0796 (5)	0.7726 (3)	0.0950 (13)	
O24	−0.3345 (4)	−0.0720 (4)	0.8274 (2)	0.0619 (9)	
H24	−0.4124	−0.0992	0.8612	0.093*	

### Atomic displacement parameters (Å^2^)

**Table:**

	*U*^11^	*U*^22^	*U*^33^	*U*^12^	*U*^13^	*U*^23^
Br1	0.0729 (4)	0.1007 (5)	0.0763 (4)	−0.0147 (3)	0.0444 (3)	0.0090 (3)
Br2	0.1079 (5)	0.0963 (5)	0.0782 (5)	−0.0219 (4)	0.0536 (4)	0.0087 (3)
N1	0.0459 (19)	0.044 (2)	0.056 (2)	0.0171 (16)	0.0223 (17)	0.0206 (18)
N2	0.0456 (19)	0.044 (2)	0.049 (2)	0.0128 (16)	0.0183 (16)	0.0164 (17)
C3	0.044 (2)	0.041 (3)	0.039 (2)	0.0135 (19)	0.0142 (19)	0.0119 (19)
N4	0.0386 (17)	0.0350 (19)	0.0361 (19)	0.0122 (15)	0.0118 (14)	0.0096 (15)
N5	0.0347 (17)	0.041 (2)	0.041 (2)	0.0145 (15)	0.0147 (14)	0.0099 (16)
C6	0.036 (2)	0.033 (2)	0.034 (2)	0.0071 (17)	0.0065 (17)	0.0032 (18)
S7	0.0403 (5)	0.0434 (7)	0.0537 (7)	0.0157 (5)	0.0182 (5)	0.0205 (5)
C8	0.037 (2)	0.039 (2)	0.038 (2)	0.0161 (18)	0.0110 (18)	0.0093 (18)
C9	0.058 (3)	0.054 (3)	0.065 (3)	0.026 (2)	0.025 (2)	0.032 (2)
C10	0.034 (2)	0.039 (2)	0.036 (2)	0.0052 (17)	0.0076 (17)	0.0027 (18)
C11	0.043 (2)	0.046 (3)	0.048 (3)	0.011 (2)	0.0139 (19)	0.014 (2)
C12	0.038 (2)	0.065 (3)	0.058 (3)	0.009 (2)	0.014 (2)	0.008 (3)
C13	0.040 (2)	0.056 (3)	0.044 (3)	−0.008 (2)	0.020 (2)	0.000 (2)
C14	0.079 (3)	0.065 (4)	0.062 (3)	0.018 (3)	0.031 (3)	0.032 (3)
C15	0.061 (3)	0.059 (3)	0.054 (3)	0.024 (2)	0.023 (2)	0.026 (2)
C16	0.057 (3)	0.039 (3)	0.046 (3)	0.002 (2)	0.018 (2)	0.004 (2)
C17	0.075 (3)	0.075 (4)	0.068 (4)	0.022 (3)	0.024 (3)	0.034 (3)
C18	0.096 (4)	0.079 (4)	0.065 (4)	0.013 (3)	0.026 (3)	0.036 (3)
C19	0.068 (3)	0.055 (3)	0.050 (3)	−0.011 (3)	0.024 (2)	0.002 (2)
C20	0.058 (3)	0.062 (3)	0.063 (3)	0.002 (2)	0.014 (2)	0.009 (3)
C21	0.057 (3)	0.055 (3)	0.055 (3)	0.004 (2)	0.014 (2)	0.014 (2)
C22	0.059 (3)	0.049 (3)	0.056 (3)	0.006 (2)	0.025 (2)	0.015 (2)
O23	0.092 (3)	0.107 (3)	0.112 (3)	0.055 (2)	0.064 (2)	0.065 (3)
O24	0.0582 (18)	0.075 (2)	0.058 (2)	0.0100 (17)	0.0270 (15)	0.0309 (18)

### Geometric parameters (Å, º)

**Table:**

Br1—C13	1.888 (4)	C12—C13	1.362 (6)
Br2—C19	1.895 (4)	C12—H12	0.9300
N1—C8	1.301 (5)	C13—C14	1.377 (6)
N1—N2	1.395 (4)	C14—C15	1.367 (6)
N2—C3	1.313 (5)	C14—H14	0.9300
C3—N4	1.359 (5)	C15—H15	0.9300
C3—C9	1.480 (5)	C16—C21	1.372 (6)
N4—C8	1.355 (4)	C16—C17	1.383 (6)
N4—N5	1.375 (4)	C16—C22	1.491 (6)
N5—C6	1.295 (5)	C17—C18	1.362 (7)
C6—C10	1.460 (5)	C17—H17	0.9300
C6—S7	1.766 (4)	C18—C19	1.364 (7)
S7—C8	1.724 (4)	C18—H18	0.9300
C9—H9A	0.9600	C19—C20	1.360 (7)
C9—H9B	0.9600	C20—C21	1.386 (6)
C9—H9C	0.9600	C20—H20	0.9300
C10—C15	1.380 (6)	C21—H21	0.9300
C10—C11	1.394 (5)	C22—O23	1.205 (5)
C11—C12	1.379 (5)	C22—O24	1.315 (5)
C11—H11	0.9300	O24—H24	0.8200
			
C8—N1—N2	104.9 (3)	C12—C13—C14	121.1 (4)
C3—N2—N1	110.0 (3)	C12—C13—Br1	119.3 (3)
N2—C3—N4	107.2 (4)	C14—C13—Br1	119.6 (4)
N2—C3—C9	126.9 (4)	C15—C14—C13	119.1 (4)
N4—C3—C9	125.9 (3)	C15—C14—H14	120.4
C8—N4—C3	106.8 (3)	C13—C14—H14	120.4
C8—N4—N5	118.7 (3)	C14—C15—C10	121.5 (4)
C3—N4—N5	134.5 (3)	C14—C15—H15	119.3
C6—N5—N4	107.6 (3)	C10—C15—H15	119.3
N5—C6—C10	122.4 (3)	C21—C16—C17	118.6 (4)
N5—C6—S7	116.7 (3)	C21—C16—C22	121.9 (4)
C10—C6—S7	120.9 (3)	C17—C16—C22	119.5 (4)
C8—S7—C6	87.70 (18)	C18—C17—C16	121.0 (5)
N1—C8—N4	111.2 (3)	C18—C17—H17	119.5
N1—C8—S7	139.6 (3)	C16—C17—H17	119.5
N4—C8—S7	109.2 (3)	C17—C18—C19	119.2 (5)
C3—C9—H9A	109.5	C17—C18—H18	120.4
C3—C9—H9B	109.5	C19—C18—H18	120.4
H9A—C9—H9B	109.5	C20—C19—C18	121.6 (4)
C3—C9—H9C	109.5	C20—C19—Br2	119.4 (4)
H9A—C9—H9C	109.5	C18—C19—Br2	119.0 (4)
H9B—C9—H9C	109.5	C19—C20—C21	118.7 (4)
C15—C10—C11	118.2 (4)	C19—C20—H20	120.6
C15—C10—C6	122.0 (4)	C21—C20—H20	120.6
C11—C10—C6	119.8 (4)	C16—C21—C20	120.7 (5)
C12—C11—C10	120.6 (4)	C16—C21—H21	119.6
C12—C11—H11	119.7	C20—C21—H21	119.6
C10—C11—H11	119.7	O23—C22—O24	123.5 (4)
C13—C12—C11	119.5 (4)	O23—C22—C16	123.2 (5)
C13—C12—H12	120.3	O24—C22—C16	113.2 (4)
C11—C12—H12	120.3	C22—O24—H24	109.5
			
C8—N1—N2—C3	−0.4 (5)	C15—C10—C11—C12	0.3 (6)
N1—N2—C3—N4	−0.1 (5)	C6—C10—C11—C12	178.6 (4)
N1—N2—C3—C9	179.6 (4)	C10—C11—C12—C13	1.1 (7)
N2—C3—N4—C8	0.5 (5)	C11—C12—C13—C14	−2.0 (7)
C9—C3—N4—C8	−179.2 (4)	C11—C12—C13—Br1	179.6 (3)
N2—C3—N4—N5	−179.2 (4)	C12—C13—C14—C15	1.6 (7)
C9—C3—N4—N5	1.1 (7)	Br1—C13—C14—C15	179.9 (4)
C8—N4—N5—C6	−0.6 (5)	C13—C14—C15—C10	−0.2 (8)
C3—N4—N5—C6	179.1 (4)	C11—C10—C15—C14	−0.7 (7)
N4—N5—C6—C10	180.0 (3)	C6—C10—C15—C14	−179.0 (4)
N4—N5—C6—S7	2.0 (4)	C21—C16—C17—C18	−0.7 (8)
N5—C6—S7—C8	−2.3 (3)	C22—C16—C17—C18	178.9 (5)
C10—C6—S7—C8	179.8 (3)	C16—C17—C18—C19	0.6 (9)
N2—N1—C8—N4	0.8 (5)	C17—C18—C19—C20	−0.4 (8)
N2—N1—C8—S7	−179.3 (4)	C17—C18—C19—Br2	179.5 (4)
C3—N4—C8—N1	−0.8 (5)	C18—C19—C20—C21	0.4 (8)
N5—N4—C8—N1	179.0 (3)	Br2—C19—C20—C21	−179.5 (4)
C3—N4—C8—S7	179.2 (3)	C17—C16—C21—C20	0.6 (7)
N5—N4—C8—S7	−1.0 (4)	C22—C16—C21—C20	−178.9 (4)
C6—S7—C8—N1	−178.3 (5)	C19—C20—C21—C16	−0.5 (7)
C6—S7—C8—N4	1.7 (3)	C21—C16—C22—O23	173.5 (5)
N5—C6—C10—C15	171.8 (4)	C17—C16—C22—O23	−6.0 (8)
S7—C6—C10—C15	−10.4 (6)	C21—C16—C22—O24	−6.7 (6)
N5—C6—C10—C11	−6.5 (6)	C17—C16—C22—O24	173.7 (4)
S7—C6—C10—C11	171.3 (3)		

### Hydrogen-bond geometry (Å, º)

**Table:**

*D*—H···*A*	*D*—H	H···*A*	*D*···*A*	*D*—H···*A*
O24—H24···N2^i^	0.82	1.87	2.674 (4)	169
C9—H9*A*···O23^i^	0.96	2.48	3.393 (6)	159

Symmetry code: (i) −*x*−1, −*y*, −*z*+2.
